# Application of soluble gas stabilization technology on ready‐to‐eat *pre‐rigor* filleted Atlantic salmon (*Salmo salar* L.)

**DOI:** 10.1111/1750-3841.16164

**Published:** 2022-05-11

**Authors:** Anita Nordeng Jakobsen, Lisa Gabrielsen, Elena Marie Johnsen, Bjørn Tore Rotabakk, Jørgen Lerfall

**Affiliations:** ^1^ Department of Biotechnology and Food Science NTNU‐ Norwegian University of Science and Technology Trondheim Norway; ^2^ Department of Processing Technology Nofima AS Stavanger Norway

**Keywords:** sustainable packaging, soluble Gas Stabilization, ready‐to‐eat salmon, salmon quality, CO_2_ solubility

## Abstract

**Abstract:**

The demand for high‐quality, convenient, and sustainable salmon products represents a potential for value‐added product development and novel packaging solutions. Soluble gas stabilization (SGS) technology, which applies dissolved CO_2_ in the product before packaging, represents a novel approach to retain product quality and prevent microbiological deterioration during cold storage of *pre‐rigor* filleted salmon loins.

The present study aimed to examine the solubility of CO_2_ in salmon loins as affected by *rigor* status. In addition, the effect of predissolved CO_2_ on the overall quality of *pre‐rigor* vacuum‐packed Atlantic salmon (*Salmo salar* L.) was investigated during storage at 4°C. The CO_2_ pretreatment was conducted, exposing loins to 100% CO_2_ for 18 h at 4°C (the control group was kept in air at 4°C) before repackaging and storage for 15 days.

Dissolved CO_2_ in the muscle (equilibrium achieved four days *post* packaging) was slightly higher in *pre‐rigor* than *post‐rigor* salmon loins (*p*_equilibrium _= 0.006). Moreover, the overall spoilage (*H*value) and microbiological stability of salmon fillets stored in SGS‐vacuum were significantly improved compared to vacuum‐packed loins (*p* < 0.05).

The results demonstrate that SGS technology can maintain the overall quality of *pre‐rigor* vacuum‐packed salmon loins without introducing the high gas‐to‐product volume ratio recognized by modified atmosphere packaging. Thus, the application of SGS technology on *pre‐rigor* loins can lead to higher economic gain and environmental benefits due to the reduced amount of required packaging material and reduced food waste.

**Practical Application:**

CO_2_ can be dissolved in *pre‐rigor* salmon loins before vacuum packaging to increase product shelf life during cold storage.

## 1 INTRODUCTION

Norwegian farmed Atlantic salmon (*Salmo salar* L.) is a highly successful product and has become a well‐known trademark globally. In 2020, Norway, as the world's largest producer of Atlantic salmon, exported 1.1 million tons with a value of €6.8 billion (FishFarmingExpert, 2021). Seventy‐seven percent of the Norwegian Atlantic salmon are exported as whole Head‐on‐Gutted (HOG) fish stored on ice. However, in Norwegian retail stores, most salmon are sold as convenient vacuum or modified atmosphere (MA) portion packages of filleted products of both *post*‐and *pre‐rigor* salmon (Heide, 2020). Products of *pre‐rigor* origin are processed before the salmon enters *rigor mortis*, which in a commercial process usually starts at 6–12 h *post‐mortem*, depending on various factors, for example, temperature, *pre‐mortem* handling, and slaughtering procedures (Chan et al., 2020; Lerfall et al., 2017; Mørkøre et al., 2008; Wang et al., 2000). *Pre‐rigor* processed salmon are regarded as a ready‐to‐eat product (sashimi and sushi quality), meaning that the producer or the manufacturer intends it for direct human consumption without the need for cooking or other processing to eliminate or reduce to acceptable level microorganisms of concern (European Commission, 2007).

The trend of consumer preferring high‐quality, convenient, and sustainable salmon products represent a potential for value‐added product development (VAPD) and increased processing before export (Dobrucka & Cierpiszewski, 2014; Nilsen, 2019). A significant advantage of *pre‐rigor* filleting is that the product can reach the market 3–5 days earlier than *post‐rigor* fillets (Skjervold et al., 2001), supplying the market with super‐fresh products. Furthermore, from a sustainability perspective, transportation of *pre‐rigor* filleted products instead of HOG will be favorable as the greenhouse gas emissions are estimated to be reduced by 20–50% (Madslien & Kwan Kwong, 2015; Rotabakk et al., 2020). Thus, effort should be put into VAPD and novel packaging techniques to establish a variety of high‐quality salmon products.

Microbiological control during storage is one of the critical factors for achieving high‐quality products. MA and vacuum packaging combined with low temperature have been used for decades to prolong the shelf life of seafood, counteracting deteriorative effects during storage due to microbiological and endogenous enzymatic activity (Bouletis et al., 2017). MA packaging utilizes the bacteriostatic effects of CO_2_ to suppress the present spoilage microbiota (Devlieghere & Debevere, 2000). Dissolved CO_2_ in the food matrix will reshape the product microbiota because of inter‐ and intraspecies variation in CO_2_ tolerance (Kolbeck et al., 2021). Growth parameters (mmax and lag phase) of Gram‐negative bacteria are more affected by CO_2_ than Gram‐positive bacteria (Devlieghere & Debevere, 2000), making gram‐positive pathogens, such as *Clostridium botulinum* and *Listeria monocytogenes* of particular concern (Jami et al., 2014; Peck et al., 2020). Furthermore, the presence of no or little O_2_ in vacuum and MA packages may promote the growth of these bacteria and the formation of botulinum neurotoxin by psychrotrophic anaerobic non‐proteolytic *C. botulinum*. The current industry practices in European countries, keeping retail vacuum‐ and MA‐packed food below 3–8°C, are supported by challenge tests for several meat products (Peck et al., 2020). However, measures toward nonproteolytic *C. botulinum* types B and F that are associated with seafood and can produce toxins at low temperature (≥3.3°C) (Peck, 2006) must be taken. Microbial growth in raw seafood is sensitive to deviations from optimal storage temperature (Hoel et al., 2017, 2018); thus, keeping an unbroken refrigerated chain during product distribution is of utmost importance.

The bacteriostatic effect of CO_2_ is proportional to the concentration of dissolved CO_2_ in the food matrix (Devlieghere et al., 1998a, 1998b). Several factors affect the amounts of dissolved CO_2_ in food, including temperature, the ratio of gas‐to‐product volume, the initial composition of the gas mixture used, and product characteristics such as pH, salt content, the content and composition of lipids, and the water content (Abel et al., 2020, 2018; Gill, 1988; Jakobsen & Bertelsen, 2004; Mendes et al., 2011; Rotabakk, 2013; Sivertsvik & Birkeland, 2006; Sivertsvik, Jeksrud et al., 2004; Sivertsvik, Rosnes et al., 2004). However, there is no available knowledge about how the *rigor‐mortis* status of salmon affects the solubility of CO_2_ in the muscle.

To achieve the optimal effect of CO_2_ in MA packaging, a gas‐to‐product volume ratio of 2:1 or 3:1 is typically used (Sivertsvik et al., 2002). From an environmental or financial point of view, this is one of the disadvantages of MA packaging. The degree of filling can be improved by combining MA‐packaging with a CO_2_ emitter (Hansen et al., 2009a) or soluble gas stabilization (SGS) technology (Sivertsvik & Birkeland, 2006). In SGS‐technology, recently reviewed by Esmaeilian et al. (2021), CO_2_ is dissolved in the product prior to packaging. Previous studies have mainly combined SGS‐technology with MA packaging (Rotabakk & Sivertsvik, 2012; Rotabakk et al., 2008). Compared to MA packaging, vacuum packaging is more transport economical and requires less packaging material. However, vacuum‐packed products are, in general, more rapidly deteriorated (Dalgaard et al., 1993; Hansen et al., 2009a; Lerfall et al., 2018a). Combining SGS‐technology and vacuum packaging represents an innovative and sustainable approach to meet the consumer demand for high‐quality fresh salmon products.

The present study aimed to (1) establish new knowledge of solubility of CO_2_ in *pre‐rigor* salmon loins and (2) evaluate the general quality and microbiological stability of *pre‐rigor* salmon loins combining *pre*‐dissolved CO_2_ and vacuum packing during cold storage.

## 2 MATERIALS AND METHODS

### 2.1 Raw material

Atlantic salmon was slaughtered at a nearby slaughterhouse in Mid‐Norway before the fish were immediately transported on ice in polystyrene (EPS) boxes to the Norwegian University of Science and Technology (NTNU, Trondheim, Norway). At arrival NTNU (5 h postslaughter), eight fish with no signs of *rigor‐mortis* was chosen and immediately scaled (average weight of 4.2 ± 0.4 kg) and filleted. The right fillets were portioned and processed directly (*pre‐rigor*), whereas left fillets were stored on ice for 4 days before further handling (*post‐rigor* samples). To ensure similar chemical composition of the experimental portions, the back‐ and mid‐loin running from the gills to the Norwegian quality cut (NQC) was used to prepare uniform experimental samples (Katikou et al., 2001; Lerfall et al., 2011).

Raw material (*n* = 5) composition was assessed by measuring fat (according to Bligh & Dyer, 1959) and water content (according to ISO.6496 (1983)), whereas the residual content was assumed to be protein and ash. Furthermore, degradation products of adenosine triphosphate (ATP) (*n* = 3), colorimetric properties (*n* = 9) and pH (*n* = 5) were analyzed as described in Section 2.4‐2.6.

### 2.2 Experimental design

**FIGURE 1:**
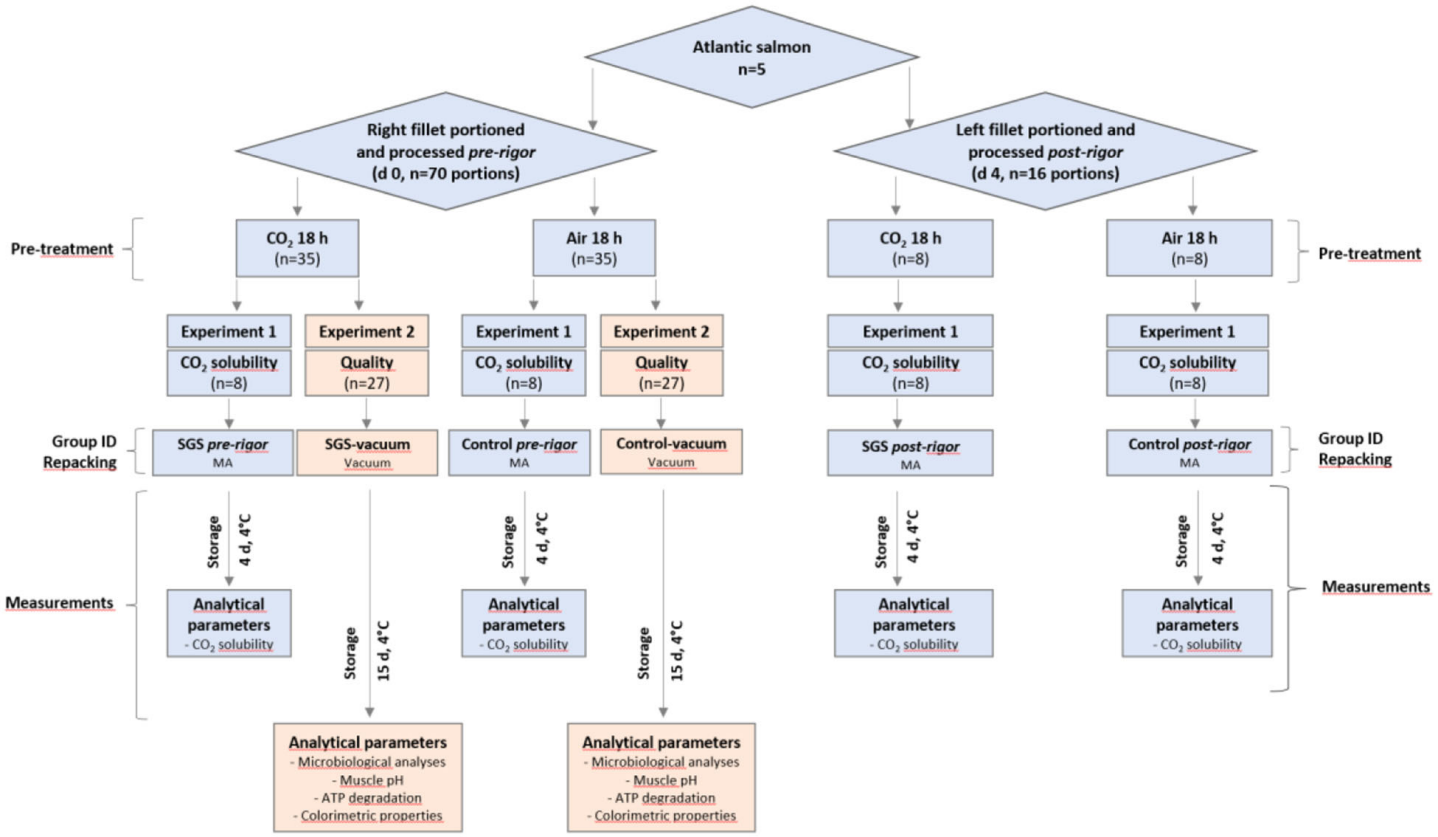
The experimental design showing the set up aiming to investigate the solubility of CO_2_ in *pre‐* versus *post‐rigor* salmon portions (Experiment 1) and to evaluate the effect of the soluble gas stabilization (SGS) technology on microbiological, chemical, and physiochemical parameters of vacuum‐packaged (25 mbar) *pre‐rigor* filleted salmon loins (Experiment 2). Modified atmosphere (MA, 60% CO_2_, 40% N_2_) packaging was used in the repacking step in experiment 1 due to the chosen methodology to measure the solubility of CO_2_ in salmon samples (Rotabakk et al., 2007)

The experimental setup was divided into two experiments (Figure 1), where Experiment 1 was designed to study the solubility of CO_2_ in portioned salmon loins as affected by the state of *rigor mortis* (*pre‐* versus *post‐rigor*). Experiment 2 followed the effect of predissolved CO_2_ on quality attributes of *pre‐rigor* vacuum‐packed salmon.

#### 2.2.1 Experiment 1: Solubility of CO_2_ in *pre*‐ and *post‐rigor* filleted salmon

In the first experiment (Figure 1), *pre‐* and *post‐rigor* samples (*n* = 8 for each group) underwent a CO_2_ pretreatment (described subsequently) for 18 h (100% CO_2_, 4°C) followed by MA‐packing (60% CO_2_ and 40% N_2_) (Group ID: SGS *pre‐rigor* and SGS *post‐rigor* respectively). The control groups were pretreated in air (18 h, 4°C), and packed in MA with the same gas composition (Group ID: control *pre‐rigor* and control *post‐rigor*, respectively). All samples (80 ± 2 g) were stored at ≤4°C for 4 days to obtain equilibrium between the headspace gas phase and product.

The CO_2_ pretreatment was performed according to Abel et al. (2020) in batches (*n* = 8). The control samples were meanwhile stored in air. After 18 hours at 4°C, pretreated samples (CO_2_ or air) were repacked into 230 ml semi‐rigid crystalline polyethylene terephthalate trays (C2125‐1A, Færch Plast, Denmark) using a tray sealing packaging machine (TL250, Webomatic, Germany). The air was evacuated (final vacuum pressure of 25 mbar) and the packages were flushed with the pre‐set MA gas mixture. The cover film comprised of a 40 mm combination of polyethylene, ethylene vinyl alcohol, polyamide, and polyethylene terephthalate (Topaz B‐440 AF, Plastopil, The Netherlands). Food‐grade CO_2_ (60%) and N_2_ (40%) were mixed using a MAP Mix 900 gas mixer (Dansensor, Denmark). The oxygen transmission rate (OTR) was 66−78 cm^3^ × 25 mm × m^−2^ × 24 h × bar at 23°C for the tray, 2.5 cm^3^ × 40 mm × m^−2^ × 24 h × atm at 23°C for the cover film, and 50 cm^3^ × m^−2^ × 24 h × bar at 23°C for the high‐barrier pouches. A sample filling degree of approximately 35% was achieved.

The headspace gas composition was measured using a Checkmate 9900 oxygen and carbon dioxide analyzer (PBI‐Dansensor, Denmark) as described by Abel et al. (2018). The gas composition was measured in empty trays immediately after packaging and in all trays at the end of the storage period (95 h). The headspace gas volume was moreover measured 2, 7, 11, 23, 35, 47, 59, 71, 83, and 95 h after packaging for the *pre‐rigor* samples and 2, 7, 11, 23, 35, 47, 60, 71, 83, and 95 h after packaging for the *post‐rigor* samples, according to a method described by Rotabakk et al. (2007) modified by Abel et al. (2018). The concentration of absorbed CO_2_ was calculated according to Rotabakk et al. (2007) related to changes in package volume as described by:

(1) $$C\begin{array}{c} t=\infty \\ CO_{2} \end{array}=\frac{1000\times P\left(V_{g}^{t=0}-V_{g}^{t=\infty }\right)\times \mathrm{Mw}CO_{2}}{R\times T\times W_{f}}$$

where $C\frac{t=\infty }{CO_{2}}$ is the total CO_2_ (ppm) absorbed by the product, *P* is absolute pressure (Pa), *V_g_* is gas volume (m^3^) at start, and at equilibrium, *Mw*CO*_2_* is the molecular weight of CO_2_, *R* is the gas constant, *T* is the absolute temperature (K), and *Wf* is the weight of the product (kg).

According to Henry's law, a sample has reached its equilibrium with the surrounding atmosphere when the amount of CO_2_ in the product headspace is proportional to the amount of CO_2_ absorbed in the sample. According to Schumpe et al. (1982) this can be described as:

(2) $$P\begin{array}{c} t=\infty \\ CO_{2} \end{array}=H_{CO_{2,p}}\times C\frac{t=\infty }{CO_{2}}$$

where $P\frac{t=\infty }{CO_{2}}$ is the partial equilibrium pressure of CO_2_ in the headspace gas (Pa), $H_{CO_{2,P}}$ is the temperature‐dependent Henry's constant for CO_2_ in the sample (Pa × ppm^−1^).

#### 2.2.2 Experiment 2: Effect of SGS‐technology on quality of *pre‐rigor* filleted vacuum‐packed salmon loins

In the second experiment (Figure 1), *pre‐rigor* filleted salmon was cut to loin pieces of 80 ± 2 g and separated into two groups. The SGS‐vacuum group (*n* = 27) was pretreated with CO_2_ (18 h, 100% CO_2_, 4°C) and the control‐vacuum group (*n* = 27) were kept in air (18 h, 4°C) as described for Experiment 1. The experiments were conducted simultaneously and with the same experimental conditions. After the pretreatment, all samples were repacked in vacuum (50 mbar), using 20‐mm PA/70‐mm PE pouches (120 × 80 mm, Star‐Pack Productive, France) and a Webomatic Supermax s3000 chamber machine (Webomatic, Germany). The OTR value of the pouches was 50 cm^3^× m^−2^ × 24 h × bar at 23°C. Three packages were opened and examined for microbiological‐, chemical‐ and physiochemical parameters at day 1, 4, 6, 8 (microbiology and pH only), 11, 13, 15 during storage at ≤ 4°C. Assessment of surface color (*n* = 3) was conducted at day 4, 11 and 15 after repacking. Samples to be analyzed for degradation products of ATP were immediately frozen at –80 ° C until analysis.

### 2.3 Microbiological analysis

A 10‐g piece of salmon was aseptically transferred to a sterile stomacher bag and diluted 1:10 with sterile peptone water (0.85% NaCl and 0.1% neutralized bacteriological peptone) and homogenized for 60 s using a Stomacher 400 lab blender (IUL Masticator, Spain). Appropriate serial dilutions were made in peptone water. Total aerobic plate count (APC), including H_2_S‐producing bacteria (black colonies), were quantified on Lyngby's iron agar (Oxoid) supplemented with 0.04% l‐cysteine (Sigma‐Aldrich, Oslo, Norway). The plates were incubated at 22°C for 72 ± 6 h.

Lactic acid bacteria (LAB) were quantified on de Man, Rogosa, and Sharp agar (MRS) (Oxoid) and incubated in an anaerobe atmosphere at 25°C for 5 days*. Brochotrix thermosphacta* was quantified on streptomycin‐thallous acetate (STA) agar containing STA selective supplement (Oxoid CM0881 and Oxoid SR0162, Oxoid Ltd., Basingstoke, UK) and incubated aerobically at 22°C for 48 ± 2 h. Microbiological indicators were selected based on previous studies of spoilage microorganisms in vacuum‐ and MA‐packed raw salmon (Food and Agriculture Organization of the United Nations (FAO), 2014; Macé et al., 2012).

### 2.4 Degradation products of ATP

Frozen samples were shredded using a kitchen grater, and approximately 1.0 g (exact weight listed) was homogenized with 7,5 ml of trichloroacetic acid (TCA, 7% w/v) for 1 min with an Ultra Turrax T25 Basic (Janke & Kunkel IKA^®^‐Labortechnik, Staufen, Germany). Potassium hydroxide was then added to the sample solution (KOH, 1 M, 3.25 mL) to achieve a pH of 6.25. The mixing tubes were kept on ice during preparation thereafter centrifuged (18000 rpm, 4°C, 15 min) in a Rotina 420R centrifuge (Hettich Zentrifugen, Germany) before the supernatant was filtered through a nylon filter (0.45 mm) and transferred to HPLC vials (Agilent, 862‐09‐16, 2 ml) for analysis.

The samples were analyzed on a Poroshell 120 porous column (ECC18 3.0 × 100 mm, porous size 2.7 mm, with a Poroshell 120 Fast Guard (3.0 × 5 mm, Sub‐2 mm), Agilent InfinityLab) after a modified method by Sellevold et al. (1986), as described by Lerfall et al. (2018a). The K value and H value were calculated based on the concentrations of ATP degradations products (Hong et al., 2017; Howgate, 2006):

(3) $$K=\frac{\left[\mathrm{Ino}\right]+\left[\mathrm{Hx}\right]}{\left[\mathrm{ATP}\right]+\left[\mathrm{ADP}\right]+\left[\mathrm{AMP}\right]+\left[\mathrm{IMP}\right]+\left[\mathrm{Ino}\right]+\left[\mathrm{Hx}\right]}\times 100\%$$

(4) $$H=\frac{\left[\mathrm{Hx}\right]}{\left[\mathrm{IMP}\right]+\left[\mathrm{Ino}\right]+\left[\mathrm{Hx}\right]}\times 100\%$$

where Ino is inosine, Hx is hypoxanthine, ATP is adenosine triphosphate, ADP is adenosine diphosphate, AMP is adenosine monophosphate, and IMP is inosine monophosphate.

### 2.5 Muscle pH

pH was measured in the center of the salmon muscle at each sampling point using a Testo 206 pH2 ‐meter (Testo SE &Co, Germany). The pH meter was calibrated before use by a two‐point calibration at pH 4 and 7.

### 2.6 Color

A digital color imaging system (DigiEye full system, VeriVide Ltd., Leicester, UK) was used to measure the surface color. The test area was manually selected, and measurements were performed above the lateral line of the fillet. Analysis was carried out in a standardized lightbox (6400 K) using a digital camera (Nikon D7000, 35 mm lens, Nikon Corp., Japan). The data obtained from the DigiEye system was processed using the software Digipix version 2.8.0.2 (VeriVideLtd.) to transform the red, green, and blue (RGB) values into the Commission Internationale de I'éclairage (CIE) values L*, a*, and b*.

### 2.7 Statistics

The data were analysed by a general linear model (GLM) with state of *rigor* and pretreatment (Air and CO_2_) as fixed factors in Experiment 1, and pretreatment (air and CO_2_) as fixed factor in Experiment 2. One‐way ANOVA tests using Tukey HSD procedures to derive statistical differences (*p* < 0.05) were used to compare groups on their respective storage days. Statistical analysis on microbial growth was done at log‐transformed data. Samples with no detected bacterial counts were scored as 1 CFU/g before log‐transformation. The bacterial counts are presented as means ± standard error (SE), and other mean values are presented as ±1 standard deviation (SD). Pearson's correlation coefficient (*r*) was used to calculate the linearity dependence between quantitative variables. Statistical analyses were performed using an IBM Statistical Package for the Social Sciences statistics software (release 26, IBM Corporation, USA). The log‐transformed average bacterial counts were fitted to the primary model of Baranyi and Roberts (1994) (available at www.combase.cc) to estimate and compare the effects of different packaging conditions on the maximum growth rates (mmax) and duration of the lag phases.

## 3 RESULTS AND DISCUSSION

### 3.1 and chemical composition of the raw material

The muscle pH at the filleting time was 7.0 ± 0.3, indicating high‐quality *pre‐rigor* loins not exposed to *pre‐mortem* stress (Lerfall et al., 2015). The average muscle fat‐ and water content were 12.1 ± 2.3% and 67.6 ± 3.0%, respectively, whereas the residual content was assumed to be protein and ash (not measured).

### 3.2 Experiment 1: Solubility of CO_2_ in *pre*‐ and *post‐rigor* filleted salmon

The solubility of CO_2_, and thus Henry's constant, depends on the product's composition (Abel et al., 2018; Jakobsen & Bertelsen, 2002; Schumpe et al., 1982; Sivertsvik, Rosnes et al., 2004). The present study assumed that the water and lipid content was similar in the *pre‐ and post‐rigor* samples (Rotabakk et al., 2018) and did not affect the experimental results. Control *pre‐rigor* packaged samples showed a significantly higher Henrys constant than the control *post‐rigor* equivalents (Table 1, 40.3±1.7 and 36.5±1.5, respectively). Moreover, the observed Henry's constants corresponded well with previously reported values for fish in general (Abel et al., 2018; Sivertsvik, Rosnes et al., 2004) and salmon specifically (Abel et al., 2020). A similar pattern was observed for the product‐ and headspace CO_2_ concentration (Table 1), showing a coincidental reduction of headspace CO_2_ with increased product concertation. However, the observed difference was only 46.3 ppm at equilibrium (*p* = 0.015), showing the practical effect of *rigor mortis* on the CO_2_ solubility as minor. However, this result implies that *pre‐rigor* CO_2_‐treatment can benefit from the increased solubility of CO_2_ and that *pre‐rigor* filleting and packaging is a highly relevant option for the salmon industry. Moreover, the SGS‐technology can be implemented as a part of the logistic chain from the slaughtering and filleting plant to a processing plant for further processing.

**TABLE 1:** Solubility of CO_2_ in salmon loins as affected by rigor mortis and pretreatment (air for the control group and CO_2_ for the SGS‐group). The CO_2_ concentration in control samples was calculated based on Equation 1 and used to calculate Henry's constant. The amount of CO_2_ in SGS samples was calculated based on Henry's constant (Equation 2)

	Control		SGS		
Parameter	*Pre‐rigor*	*Post‐rigor*	*Pre‐rigor*	*Post‐rigor*	*p*‐Value*
Product CO_2_ [ppm] _initial_	–	–	1479 ± 136^a^	1224 ± 46^b^	<0.001
Product CO_2_ [ppm] _equilibrium_	1014 ± 29^d^	1060 ± 33^c^	1487 ± 21^a^	1459 ± 11^b^	<0.001
Headspace CO_2_ [%] _equilibrium_	41.4 ± 0.2^c^	38.2 ± 0.2^d^	60.7 ± 0.9^a^	58.0 ± 0.5^b^	<0.001
Henry's constant [Pa/ppm]	40.3 ± 1.7^a^	36.5 ± 1.5^b^			<0.001

*Note*: Superscript letters indicate a significant difference at level *α* = 0.05 within each row.

* GLM + TukeyHSD, *n* = 8.

The CO_2_ solubility observed in the SGS *pre‐rigor* and SGS *post‐rigor* samples are presented in Table 1 as the initial CO_2_ product concentration at the point of repackaging and as solubilized CO_2_ at equilibrium after storage in a MA consisting of 60% CO_2_ and 40% N_2_. Similar to the headspace CO_2_ concentration (Table 1), significantly higher CO_2_ concentration was observed in the SGS‐groups as compared to the controls (GLM, *p* < 0.001). Moreover, the effect of *rigor mortis* was significant, showing higher amounts of dissolved CO_2_ in *pre‐rigor* compared to *post‐rigor* samples (Table 1, *p*_initial _< 0.001 and *p*_equilibrium _= 0.006).

**FIGURE 2:**
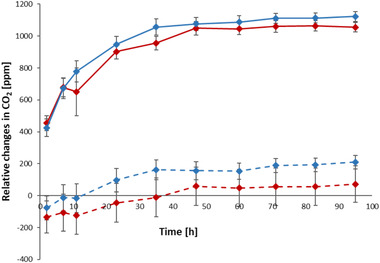
Relative changes in dissolved CO_2_ (ppm) as affected by time, *rigor mortis* (*pre*‐ versus *post*‐*rigor* filleted salmon loins), and pretreatment (air for the control group or CO_2_ for the SGS group). Presented values are based on the difference between the initial concentration and the dissolved concentration at the measure time (h). Legends: 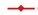. Control *pre‐rigor*, 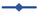. Control *post‐rigor* 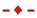. SGS *pre‐rigor*, 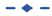. SGS *post‐rigor*

Relative changes in product CO_2_ concentration in salmon loins as a function of storage time are presented in Figure 2. The SGS *pre‐rigor* and SGS *post‐rigor* groups had a significantly lower change in dissolved CO_2_ than the control samples (GLM, *p *< 0.001), as expected, as CO_2_ has already been dissolved into the product during the CO_2_ pretreatment step, bringing the system closer to the new equilibrium. Figure 2 shows that 50% of the equilibrium concentration is achieved between 4 and 7 h for the control samples, showing that a significant amount of CO_2_ is dissolved quickly, enabling CO_2_ as a pretreatment before repackaging in vacuum, MA, and further distribution to the market. The effect of *rigor mortis* on the relative change in product CO_2_ concentration among the control samples was insignificant. However, among the SGS samples, an effect of *rigor mortis* was observed, showing significantly less dissolved CO_2_ in *post‐rigor* than *pre‐rigor* samples (*p* < 0.05).

At equilibrium (*t* > 47 h), it was assumed that the *pre‐rigor* treated samples were in a *post‐rigor* state, which is supported by Mørkøre et al. (2008), who reported stressed Atlantic salmon to be in full *rigor* within 36 h. Moreover, Wang et al. (2000) reported from their study that *rigor mortis* started 8 h after death and that it achieved full *rigor* after 24–60 h before *rigor* was wholly dissolved after 72 h. The results obtained in experiment 1 indicate better solubility in *pre‐rigor* than *post‐rigor* Atlantic salmon portions. As far as we know, this is the first study that has investigated the CO_2_ solubility in *pre‐rigor* Atlantic salmon. The findings highlight the potential of SGS‐technology for an industrial application for both *pre*‐ and *post‐rigor* salmon loins.

### 3.3 Experiment 2: Effect of SGS‐technology on quality of *pre‐rigor* filleted vacuum‐packed salmon loins

A second experiment was designed to investigate further the SGS technology's potential to retain quality attributes of high‐end cuts of *pre‐rigor* filleted vacuum‐packed salmon. Quality parameters were evaluated during 15 days of refrigerated storage and compared to vacuum‐packed portions. For both groups, the muscle pH dropped significantly between filleting and storage day 1 (GLM, *p* <0.001), that is, from pH 7.0 ± 0.3 to 6.3 ± 0.08 for the SGS‐vacuum samples and 6.4 ± 0.04 for the control‐vacuum samples. The mean pH of the samples remained stable through the storage period.

The CO_2_‐pretreatment of the SGS‐samples resulted in a significantly lower mean muscle pH of 6.3 ± 0.07 throughout the storage period than for the control‐vacuum group with a mean pH value of 6.4 ± 0.06 (GLM, *p* < 0.005). CO_2_ lowers the food matrix's pH due to dissociation into carbonic acid when reacting with water (Sivertsvik et al., 2002). Thus, similar pH‐drops due to CO_2_ dissolutions have been reported for different kinds of food products such as pork loins (Sørheim et al., 1996), Atlantic halibut (Rotabakk et al., 2008), and chicken breast (Rotabakk et al., 2006).

#### 3.3.1 Microbiological stability

The initial concentration of APC in the *pre‐rigor* salmon loin was below the method quantification limit of 2.4 log CFU × g^−1^ (Nordic Committee on Food Analysis, 2006), indicating that contamination during handling and processing was at a minimum. The low initial microbiological contamination level is in accordance with previous studies of *pre‐rigor* salmon loin quality (Hanesen et al., 2009; Lerfall et al., 2018b). Several bacterial genera can evolve in MA‐packed salmon during storage and contribute to spoilage, including genera of LAB such as Carnobacterium spp., *Brochothrix thermosphacta*, and gram‐negative species capable of anaerobic respiration, including, *Photobacterium phosphoreum*, psychotropic Enterobacteriaceae, and *Shewanella putrefaciens* (Gram & Huss, 1996; Macé et al., 2012; Powell & Tamplin, 2012). The application of dissolved CO_2_ in the food matrix is a hurdle in food preservation that reshape the microbiota initially present because of inter‐ and intraspecies variation in CO_2_ tolerance (Kolbeck et al., 2021).

**FIGURE 3:**
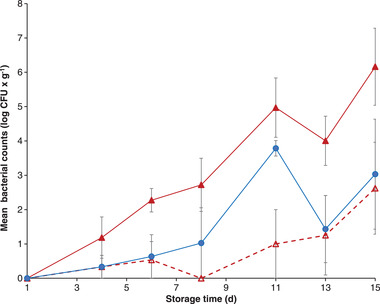
Evolution of total aerobic plate counts (APC) and H_2_S‐producing bacteria of *pre‐rigor* vacuum‐packed Atlantic salmon loins pretreated with CO_2_ (SGS‐vacuum) or air (control‐vacuum) during storage at 4°C. Each sampling point represents the mean bacterial count (*n* = 3) ± SE. Legends: 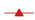. APC, control‐vacuum; 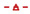. APC, SGS‐vacuum; 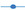. H_2_S‐producing bacteria, control‐vacuum

The evolution of APC was significantly inhibited in the SGS vacuum‐packed samples compared to the control group (Figure 3, GLM, *p* < 0.0001), reaching a maximum level of 2.6 ± 1.3 and 6.2 ± 1.1 log CFU × g^−1^ respectively, after 15 days of cold storage. There was no correlation between the APC and the measured muscle pH (*r* = 0.27 and *r* = 0.012 SGS‐vacuum and control‐vacuum samples, respectively). Although a randomized experimental design was conducted, high biological variation was observed (seen as high SE of microbiological counts), indicating a nonuniform distribution of microorganisms due to, for example, point contamination of the fillets during processing or atmosphere leakage in the packages during storage. The packages were controlled before analysis, so the uneven distribution of fillet microbiota (concentration and community structure) is likely the reason. Thus, attention should be given to the overall picture, not on specific sampling days.

There are no specific criteria for APC of RTE raw salmon loins, but several guidelines and studies state APC levels > 6 log CFU × g^−1^ as borderline or unsatisfactory quality (Food Safety Authority of Ireland, 2020; Gilbert et al., 2000). The estimated duration of the lag‐phase by the primary model of Baranyi and Roberts was 0.9 ± 2.6 days for APC in the control‐vacuum group, which increased to 9.7 ± 1.1 days for SGS‐vacuum. Thus, the CO_2_ pretreatment can be an efficient hurdle to delay microbial growth, similar to previously reported for *pre‐rigor* filleted Atlantic salmon packed in MA with or without a CO_2_ emitter (Hansen et al., 2009a). The lag‐phase extension depends on the initial microbiota present and the amount of dissolved CO_2_ in the food matrix as Gram‐negative spoilage bacteria, in general, are more influenced by the concentration of dissolved CO_2_ than Gram‐positive bacteria (Devlieghere & Debevere, 2000). Furthermore, Devlieghere and Debevere (2000) established an inverse relationship between the concentration of dissolved CO_2_ and the lag phase for common spoilage microorganisms.

After microbiological growth was initiated in the present study, the estimated maximum growth rate of APC was similar under the two packaging conditions applied (*m*_max_ 0.41 ± 0.07 day^−1^ and m_max_ 0.43 ± 0.11 day^−1^ for control‐vacuum and SGS‐vacuum samples, respectively). Contradictory, SGS packaging in combination with conventional heat treatment (Abel et al., 2019) or microwave heat treatment (Lerfall et al., 2018b) approximately halved the maximum growth rate of APC compared to heat treatment of vacuum‐packed salmon. However, the latter study reported no effect of CO_2_ emitters on the growth rate of APC compared to vacuum. Devlieghere and Debevere (2000) established a linear relationship between dissolved CO_2_ in the food matrix and mmax of selected spoilage bacteria; however, as APC reflects the total microbial community, it cannot be compared directly.

H_2_S‐producing bacteria was not detected in the raw material. Pretreatment with CO_2_ resulted in no detectable H_2_S‐producing bacteria, whereas low counts (<3.8 log CFU × g^−1^) were detected from day four and throughout the storage period for the control‐vacuum group (Figure 3). H_2_S‐producing bacteria like *Shwewanella putrefaciens* is known to be CO_2_‐sensitive (Boskou & Debevere, 1998; Dalgaard, 1995). The CO_2_‐sensitivity of *S. putrefaciens* has been confirmed in MA‐packages of for example, cod (Hansen et al., 2007) and saith (Lerfall et al., 2018a). The result also agrees with the previous results of Hansen et al. (2009b), which detected low counts of H_2_S‐producing producing bacteria in MA‐packed salmon. Boskou and Debevere (1998) reported that a pH‐drop of 0.2 units, from 6.4 to 6.2, increased the lag‐phase of *S. putrefaciens* in MA‐packed cod. Thus, the pH difference between the groups in the presented study might also influence the growth of H_2_S‐producing bacteria. Fuentes‐Amaya et al. (2016) demonstrated the ability of H_2_S‐producing bacteria to proliferate in vacuum‐packed Atlantic salmon at 4°C, thus including SGS‐technology as an extra barrier can prevent spoilage due to the growth of this bacteria group.

A significantly higher LAB concentration (GLM, *p* = 0.016) was observed for the control‐vacuum samples than the SGS‐vacuum samples during storage, reaching a final concentration at day 15 of 6.2 ± 1.1 log CFU × g^−1^ and 2.0 ± 2.0 log CFU × g^−1^, respectively. Lactic acid bacteria were not detected in the first 6 days of storage in neither group, but overall, a significant correlation between the concentration of APC and LAB was found for the control‐vacuum group (*p* < 0.01, *r* = 0.896). Application of MA‐ and vacuum packaging selects for growth of LAB (Gram & Dalgaard, 2002). However, in the present study, we aimed to inhibit the development of LAB with the CO_2_ pretreatment. The same phenomenon was reported by (Hansen et al., 2009a) by combining MA packaging and CO_2_ emitters for *pre‐rigor* Atlantic salmon fillets.

*B.thermosphacta*, a common spoilage bacteria in MA‐ and vacuum‐packed fish and meat products (Macé et al., 2012; Mamlouk et al., 2012; Stanborough et al., 2017), was only detected in the control‐vacuum samples at day 15 with a count of 2.3 ± 1.2 log CFU × g^−1^ indicating that CO_2_ pretreatment of vacuum‐packed salmon loins can be beneficial to prevent the growth of this spoilage bacteria. *B. thermosphacta* are also previously described to be inhibited by dissolved CO_2_ in the food matrix, although it is a Gram‐positive bacteria (Devlieghere & Debevere, 2000). The use of packaging atmosphere with less O_2_ and more CO_2_ has previously been proposed to reduce the spoilage potential of *B. thermosphacta* in MA‐packed shrimps.

#### 3.3.2 ATP degradation products

Fish deterioration and loss of seafood quality can be monitored by analyzing ATP degradation products (Hong et al., 2017; Howgate, 2006). Endogenous and bacterial enzymes catalyze *post‐mortem* degradation of ATP in the fish muscle through the intermediate products ADP, AMP, IMP, Ino, and Hx. Inosine monophosphate is associated with fish freshness and the pleasant umami taste (Hong et al., 2017), and effort should be made to maintain the freshness of the IMP level in seafood. The further degradation from Ino to Hx result in the development of unpleasant flavors in stored fish (Hong et al., 2017; Howgate, 2006).

**FIGURE 4:**
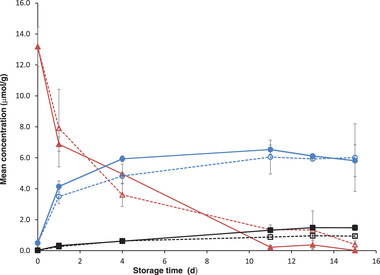
Mean concentration (*n* = 3) of Inosine monophosphate (IMP), Inosine (Ino), Hypoxanthine (Hx) in *pre‐rigor* vacuum‐packed Atlantic salmon loins pretreated with CO_2_ (SGS‐vacuum) or air (control‐vacuum) during storage at 4°C. Legends: 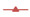. IMP, control‐vacuum; 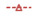 IMP, SGS‐vacuum; 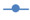 Ino, control‐vacuum; 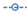 Ino, SGS‐vacuum; 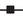 Hx, control‐vacuum; 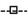 Hx, SGS‐vacuum

In the present study, the samples showed a significant drop in the concentration of IMP and a rapid increase in Ino level between storage days 0 and 4 regardless of packing (Figure 4). The highest concentration of Ino was found at day 11 for both groups, at the time point where IMP was depleted in the control‐vacuum samples. The IMP concentration was significantly higher in the SGS‐vacuum samples than the control‐vacuum samples at storage days 11 and 15 (One‐way ANOVA, *p* = 0.004 and *p* = 0.047, respectively). Findings from the present study indicate that SGS‐technology prolongs the freshness of *pre‐rigor* vacuum‐packed salmon loins by slowing down the degradation of IMP to Ino.

A significant effect of the packaging conditions was found on the muscle concentration of Hx (GLM, p = 0.05). Between storage days 0 and 4, the value was low in both groups (Figure 4), however from day 11, the concentration was higher in the control‐vacuum samples than in the SGS‐vacuum samples. On day 15, a maximum concentration of 1.48 and 0.94 mmol/g of Hx was reached in the control‐vacuum and SGS‐vacuum samples, respectively. The results are consistent with the significantly higher APC level in control‐vacuum than SGS‐vacuum samples, as Hx formation is catalyzed by both endogenous fish enzymes and bacterial enzymes (Hong et al., 2017). A significant correlation was found between the Hx concentration and the level of APC, H_2_S‐producing bacteria, IMP and Ino (*r* = 0.842, *r* = 0.564, *r* = −0.848, *r* = 0.673, respectively, *p* < 0.01)

**FIGURE 5:**
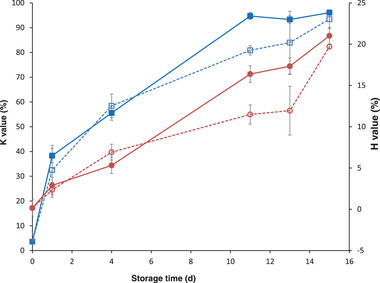
Mean (*n* = 3) K and H values during cold storage of *pre‐rigor* vacuum‐packed Atlantic salmon loins pretreated with CO_2_ (SGS‐vacuum) or air (control‐vacuum) during storage at 4°C. Legends: 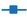 K value, control‐vacuum; 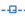 K value, SGS‐vacuum; 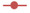 H value, control‐vacuum; 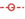 H value, SGS‐vacuum

The *K* value is used to evaluate the quality of raw fish freshness before significant microbiological spoilage is initiated (Hong et al., 2017). The raw material of day 0 had a low *K* value of 3.6 ± 0.4%, indicating fish of superior quality as the final degradation products of ATP (In and Hx) are barely present (Figure 5). On day 11, the *K* value in control‐vacuum samples reached 94.7 ± 1.4%, significantly higher than the SGS‐vacuum samples at 81.7 ± 1.7% (One‐way ANOVA, *p* = 0.003).

Japanese researchers have suggested a *K* value of 20% as a limit for raw fish of sashimi quality (Hamada‐Sato et al., 2005). However, Erikson et al. (1997) suggested salmon with *K* values lower than 50% to be of excellent quality. *K* value as a freshness indicator is disputed as it is highly dependent on species, and external factors such as season, handling conditions, and method of killing (Hong et al., 2017)

*H* value is a quality parameter used to evaluate seafood spoilage as it reflects the amount of Hx accumulated. Hx are formed from the autolytical breakdown of ATP and produced by spoilage microorganisms (Hong et al., 2017). In the present study, the SGS‐technology resulted in significantly lower *H* values at day 11 and 13 (One‐way ANOVA, *p* = 0.011 and *p* = 0.046 respectively), reaching 11.4 ± 1.1% and 16.4 ± 0.84% for SGS‐vacuum and control‐vacuum samples respectively at day 11. In addition, a significant correlation was found between *H* value and APC for both groups (*r* = 0.804 and *r* = 0.931 for SGS‐vacuum and control‐vacuum, respectively), indicating a microbial contribution to the Hx formation.

#### 3.3.3 Color

*Pre‐rigor* filleted RTE salmon is regarded as a high‐quality sashimi product. The appearance is therefore of high importance to the consumers. Color is generally perceived as one of salmon fillets’ most important quality parameters (Anderson, 2001). The price per kg of salmon is positively correlated to fillet redness (Haegermark, 2012). Thus, color deviations of products in new packaging compared to the conventional product can be unfavorable as they affect the consumers’ perception of the product and willingness to pay (Alfnes et al., 2006). For the present experiment, the coulometric variables L*, a*, and b* with respect to storage day and packaging conditions are given in Table 2. The flesh appearance changed after packing. The samples were lighter (higher L*‐value) and more reddish (higher a*‐value) at the first sampling point than the raw material at day 0. However, no differences in the flesh appearance were found between the SGS‐vacuum and the control‐vacuum samples. Furthermore, the storage time did not affect the visual appearance of the samples between days 4 and 15 of storage. Hence, the increased amount of CO_2_ due to the CO_2_ pretreatment did not pose any visual effect. Contradictory, Hansen et al. (2009a) found MA‐packed Atlantic *pre‐rigor* salmon with and without CO_2_ emitters to have more redness and yellowness after 15 days of cold storage than vacuum‐packed samples. Chan et al. (2021) reported that MA‐packed (60% CO_2_, 40% N_2_) salmon fillets were darker, more reddish, and yellowish than vacuum skin‐packed fillets. Choubert and Baccaunaud (2006) demonstrated that a gas mixture composed of CO_2_:N_2_ (40:60) preserved the color of the trout fillets to a greater extent than air during 3‐week refrigerated storage. On the other side, bleaching of fillets may occur during storage in high headspace CO_2_ concentrations (90%) (Barnett et al., 1982). Most of these studies do, however, report color changes in MA‐packaged salmon portions allowing the surface to dry during storage. In the present study, this was not the case since the packaging film was in contact with the product maintaining the surface humidity and thereby giving better protection of the color of the products during storage.

**TABLE 2:** Color parameter (L*, a*, and b*) values of pre‐rigor vacuum‐packed salmon loins during 15 days of refrigerated storage at 4°C (*n* = 3 for packed samples, *n* = 9 for raw material at day 0). The loins were pretreated with air (control‐vacuum) or CO_2_ (SGS‐vacuum) for 18 h prior to packaging

Group	Storage day	Control‐vacuum	SGS‐vacuum	*P*_G_	Main effect^2^
*L* ^*^	0^1^	61.5 ± 0.8^a^	61.5 ± 0.8^a^	–	0.344
	4	64.9 ± 2.6^b^	66.1 ± 1.2^a,b^	0.51	
	11	66.0 ± 1.4^b^	67.1 ± 1.2^c^	0.36	
	15	64.2 ± 1.4^a,b^	64.3 ± 1.4^b^	0.94	
	*P*_D_	0.001	<0.001		
*a* ^*^	0^a^	17.7 ± 0.9^a^	17.7 ± 0.9^a^	–	0.45
	4	19.0 ± 1.4^a,b^	19.4 ± 1.0^a^	0.69	
	11	19.9 ± 1.2^b^	19.4 ± 1.6^a^	0.63	
	15	18.9 ± 0.6^a,b^	17.7 ± 0.6^a^	0.09	
	*P*_D_	0.018	0.029		
*b* ^*^	0^a^	14.9 ± 0.7^a^	14.9 ± 0.7	–	0.37
	4	15.6 ± 0.9^a^	15.5 ± 0.5	0.94	
	11	15.3 ± 0.7^a^	14.7 ± 0.7	0.34	
	15	15.9 ± 0.3^a^	15.7 ± 0.3	0.50	
	*P*_D_	0.18	0.14		

*Note*: Statistical differences were calculated using General Linear Model (GLM) analyses of variance. *P*_G_‐values and *P*_D_‐values are the significance level for the effect of packaging technology at each sampling point and storage time within each group, respectively. Different superscripts indicate significant variation (GLM, *p* < 0.05, Tukey HSD) within a group due to storage time.

^1^ Results for the control‐vacuum and SGS‐vacuum groups at day 0 are equal and represent the raw material.

^2^ Overall comparison of control‐vacuum and SGS‐vacuum packaging (GLM) without data of the raw material.

## 4 CONCLUSION

The present study demonstrated significantly higher amounts of dissolved CO_2_ in *pre‐rigor* than in *post‐rigor* Atlantic salmon loins. However, the practical effect of the increased CO_2_ solubility in a real‐world application is probably of minor significance. In addition, favorable effects of SGS‐treatment in combination with vacuum packing of *pre‐rigor* loins of salmon stored at 4°C were demonstrated. The SGS‐technology resulted in lower growth rates of APC, total inhibition of H_2_S producing bacteria, and slower formation of Hx compared to the control group. Furthermore, no significant effect on the color appearance of the salmon filets due to the SGS‐treatment was found. The results demonstrate that SGS technology combined with vacuum packaging is suitable for preserving high‐quality *pre‐rigor* filleted salmon products. The novel packaging solution represents a high industrial potential for value‐added product development for a broader market.

## AUTHOR CONTRIBUTIONS

Anita Nordeng Jakobsen: Conceptualization; Data curation; Formal analysis; Funding acquisition; Investigation; Methodology; Project administration; Supervision; Writing – original draft; Writing – review & editing. Lisa Gabrielsen: Data curation; Formal analysis; Investigation; Methodology; Writing – review & editing. Bjørn Tore Rotabakk: Conceptualization; Data curation; Formal analysis; Funding acquisition; Investigation; Methodology; Project administration; Supervision; Writing – review & editing. Jørgen Lerfall: Conceptualization; Data curation; Formal analysis; Funding acquisition; Investigation; Methodology; Project administration; Supervision; Writing – original draft; Writing – review & editing.

## CONFLICTS OF INTEREST

The authors declare no conflicts of interest.
